# A Sensitive Branched DNA HIV-1 Signal Amplification Viral Load Assay with Single Day Turnaround

**DOI:** 10.1371/journal.pone.0033295

**Published:** 2012-03-27

**Authors:** Mark A. Baumeister, Nan Zhang, Hilda Beas, Jesse R. Brooks, Jesse A. Canchola, Carlo Cosenza, Felix Kleshik, Vinod Rampersad, Johan Surtihadi, Thomas R. Battersby

**Affiliations:** Siemens Healthcare Diagnostics Inc., Berkeley, California, United States of America; University of Houston, United States of America

## Abstract

Branched DNA (bDNA) is a signal amplification technology used in clinical and research laboratories to quantitatively detect nucleic acids. An overnight incubation is a significant drawback of highly sensitive bDNA assays. The VERSANT® HIV-1 RNA 3.0 Assay (bDNA) (“Versant Assay”) currently used in clinical laboratories was modified to allow shorter target incubation, enabling the viral load assay to be run in a single day. To dramatically reduce the target incubation from 16–18 h to 2.5 h, composition of only the “Lysis Diluent” solution was modified. Nucleic acid probes in the assay were unchanged. Performance of the modified assay (assay in development; not commercially available) was evaluated and compared to the Versant Assay. Dilution series replicates (>950 results) were used to demonstrate that analytical sensitivity, linearity, accuracy, and precision for the shorter modified assay are comparable to the Versant Assay. HIV RNA-positive clinical specimens (n = 135) showed no significant difference in quantification between the modified assay and the Versant Assay. Equivalent relative quantification of samples of eight genotypes was demonstrated for the two assays. Elevated levels of several potentially interfering endogenous substances had no effect on quantification or specificity of the modified assay. The modified assay with drastically improved turnaround time demonstrates the viability of signal-amplifying technology, such as bDNA, as an alternative to the PCR-based assays dominating viral load monitoring in clinical laboratories. Highly sensitive bDNA assays with a single day turnaround may be ideal for laboratories with especially stringent cost, contamination, or reliability requirements.

## Introduction

Branched DNA (bDNA) technology, first developed over twenty years ago [1], is today widely used in clinical [2] and research [3] laboratories to quantitatively detect specific nucleic acid sequences. bDNA quantitative hybridization technology has a wide dynamic range and is sensitive enough for applications intended to reliably detect very few target molecules [4]. The technology has been described in detail elsewhere [4]. bDNA technology has some advantages over PCR technology, commonly used for applications requiring high sensitivity. Unlike PCR, where a region of the intended target is exponentially amplified in order to generate detectable signal, in bDNA assays only signal is amplified. bDNA assays are consequently not susceptible to contamination risks associated with PCR-based assays [5]. Minor sequence variation in probe-binding regions of target will not compromise assay performance, in contrast to PCR [6], leading to more robust quantitation across genotypes [7]. Furthermore, reproducibility, and therefore reliability, of bDNA assays is superior to PCR assays, especially at the low end of an assay's dynamic range [8].

However, a reduction in the processing time allowing the single day turnaround now standard in clinical PCR assays, would improve the productivity of laboratories utilizing bDNA. bDNA is a hybridization technology in which a series of probes is introduced to a sample well. Target is immobilized at the well surface and an extended branching structure with many signal-generating moieties is constructed to produce an amplified signal [4]. The primary component of the run time is an overnight (16–18 h) target incubation. Target incubation involves annealing of unpurified lysed target nucleic acids to a set of probes used in immobilizing the target and another set of probes to initiate construction of the extended signaling structure. We have modified the Versant HIV-1 RNA 3.0 Assay (bDNA) (“Versant Assay”) currently in use in clinical laboratories to allow the viral load assay to be run in a single day (assay in development; not commercially available). An evaluation of the modified assay consisting of analytical performance testing, a method comparison with clinical specimens, a genotype equivalence study, and testing of potentially interfering endogenous substances is presented.

## Results

In an initial experiment we simply shortened the standard 16–18 h target incubation to a target period of 3 h, forcing the otherwise unmodified Versant Assay to provide results in little over a standard 8 h work shift. We examined analytical performance of the unmodified shortened assay with a target dilution series of non-infectious HIV-1_8E5/LAV_ virus (8E5) [9]. Performance suffered when comparing analogous parameters previously reported [2] for the Versant Assay. Assay sensitivity and precision were significantly worse with a simple truncation of the target incubation (Table S1).

Therefore, we focused on accelerating signal generation in the assay by speeding the diffusion of the ∼10 Kb target HIV RNA to the well surface and accelerating hybridization of the assay probes and target. Dilution series of target were analyzed to assess the impact of assay modification in these evaluations. Diffusion is most simply addressed by reducing the volume in the assay wells during target incubation. However, there are at least two potential problems with this approach. Surfaces of the wells have attached capture probe oligonucleotides. When the assay solution volume is reduced, some capacity for signal generation is inevitably lost as the solution level falls below some of the capture oligonucleotides. Additionally, sufficient solution must be present in the wells to ensure that the small volume lost to evaporation during assay manipulations does not significantly affect assay results.

**Figure 1:**
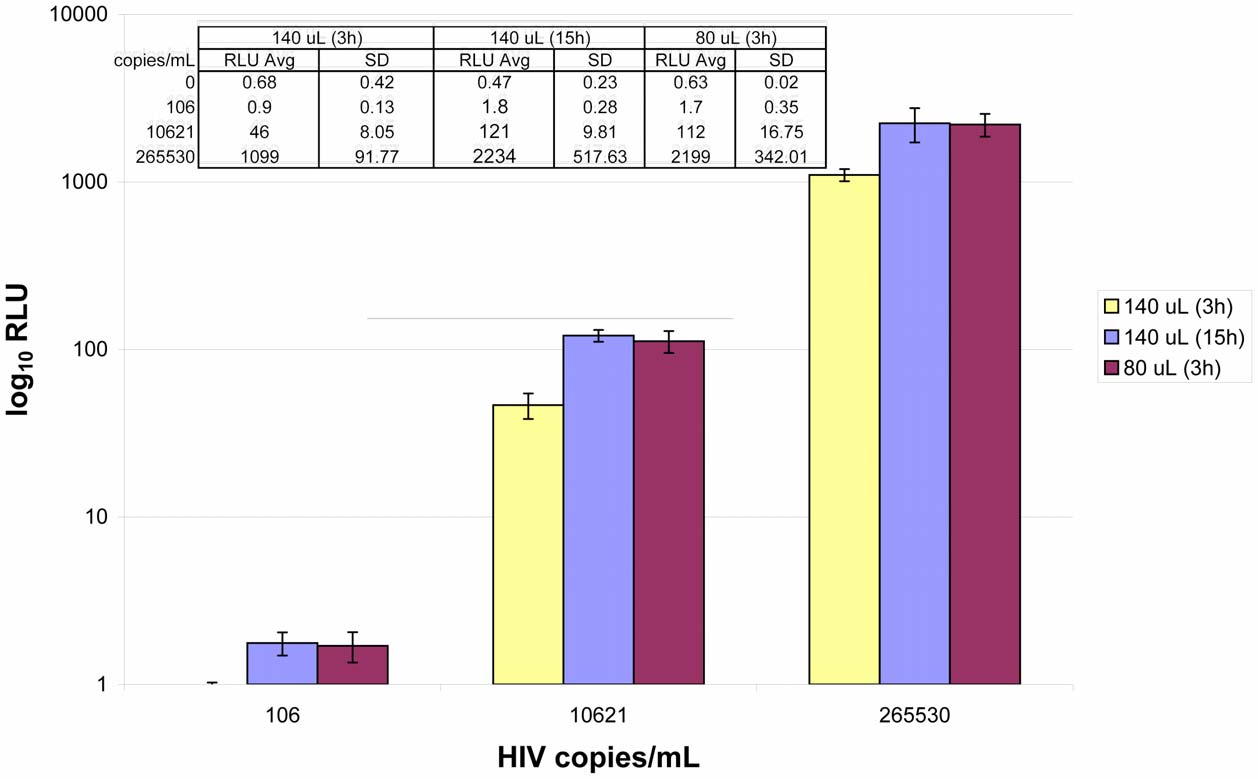
Signal generation in standard and low volume assays. The standard assay was run with 3 and 15 h target incubation (140 µL) and the low volume assay was run with 3 h target incubation (80 µL). Identical lots of all components except Lysis Diluent were used on a single instrument for the three conditions. A dilution series of 8E5 in Seracon II at three concentrations (n = 10 for each condition) generated nearly identical average Relative Light Units (RLUs) for standard volume target incubation at 15 h and low volume target incubation volume at 3 h. Signal in negative samples remained unchanged (n = 6 for each condition).

Reducing the target incubation solution volume as low as 80 µL still left sufficient solution in the wells such that evaporation was not a problem under the assay conditions. Signal generation at 80 µL target incubation solution volume was much more rapid. Salt and surfactant concentrations in the solution at the lower volume were also adjusted to give rapid signal generation without proportionally increasing background signal in negative samples. Doubling these concentrations in the “Lysis Diluent” component gave optimal results for the lower volume target incubation (Table S2). Signal at 3 h with this modified, “low volume” condition was virtually identical to signal at 15 h with the standard Lysis Diluent volume across the quantitative range of the assay (Fig. 1).

**Figure 2:**
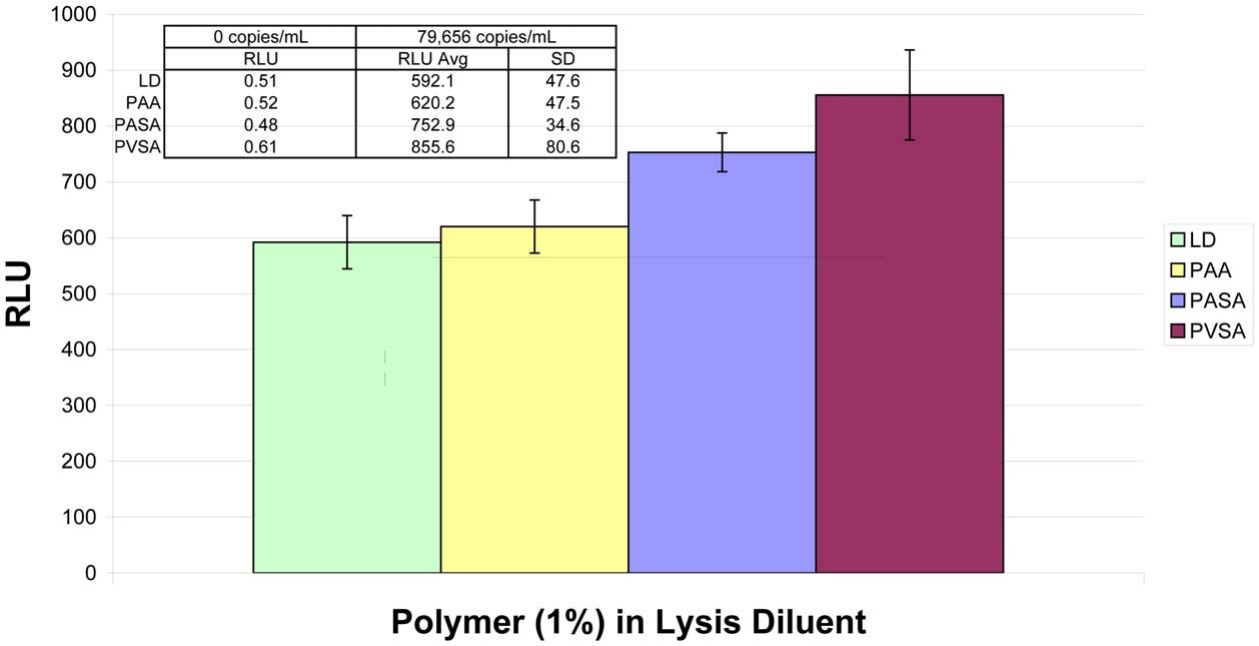
Polyanions in target incubation solution. In this example, addition of 1% (w/V) of polyacrylic acid (PAA), polyantholesulfuric acid (PASA), or polyvinylsulfonic acid (PVSA) to the Lysis Diluent (LD, control) caused different enhancement of signal generation relative to the control condition. An 8E5 sample at 80,000 copies/mL (n = 5 for each condition) was analyzed here on a single plate with 3 h target incubation. Superior signal enhancement by PVSA relative to alternative polyanions was observed throughout our experiments. No polyanion caused discernable increase in signal with negative samples (n = 1 for each condition).

**Figure 3:**
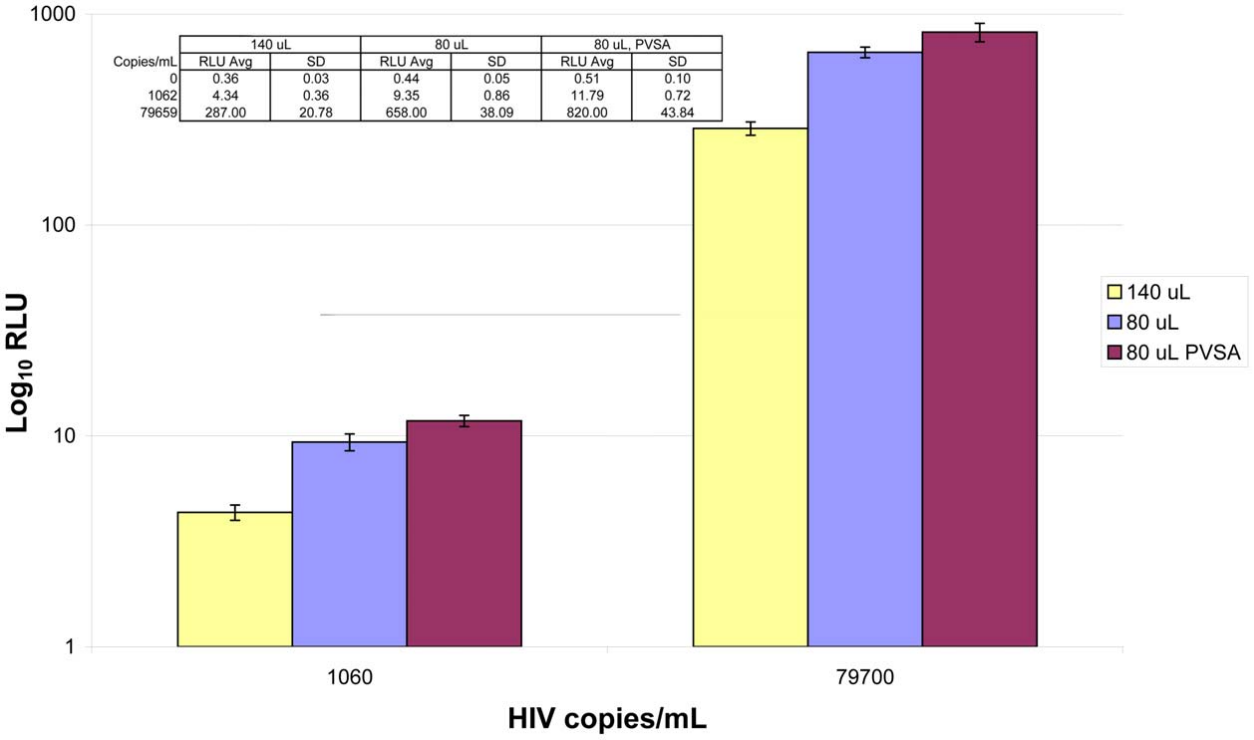
Cumulative effect of lower volume and PVSA addition. Three conditions were compared in this experiment: Versant HIV-1 RNA 3.0 Assay with standard volume target incubation (140 µL), low volume target incubation (80 µL), or low volume target incubation plus PVSA (80 µL, PVSA). Two concentrations of 8E5 (n = 4 for each level of each condition) were analyzed on a single plate with 2 h target incubation. Enhanced signal generation (RLU average) was observed for low volume target incubation, and further improvement was observed upon addition of PVSA to the low volume target incubation. Although signal increased slightly for negative samples (n = 6 for each condition) in the low volume conditions, signal for the low concentration positive samples in the low volume conditions increased more than enough to compensate.

Various polyanions were tested for ability to accelerate nucleic acid hybridization [10] in the assay (Fig. 2). Several polyanions that were examined significantly accelerated signal generation in the assay. Among the polyanionic species in our experiments, polyvinylsulfonic acid (PVSA) was consistently superior in rapidly boosting assay signal across the quantitative range of the assay. Optimization of PVSA concentration in the assay demonstrated that 0.3% (w/v) PVSA in the Lysis Diluent significantly increased signal generation, yet did not cause problems with precipitation upon storage (data not shown). Finally, the lower volume Lysis Diluent was prepared with PVSA (designated “M1 Lysis Diluent”) and used in assays with low volume target incubation, demonstrating that the two modifications together had some cumulative benefit in rapid signal generation (Fig. 3).

Preliminary experiments indicated that the levels of signal generated in the Versant Assay could be reached in as little as 2.5 h target incubation time simply by using M1 Lysis Diluent in the assay. Probe-containing components and associated volumes of addition were not changed. Assay evaluations with the M1 Lysis Diluent and assay software modified to accommodate 2.5 h target incubation (the “modified assay”) were performed, including analytical performance testing, a method comparison with clinical specimens, a genotype equivalence study, and testing of potentially interfering endogenous substances.

Using data gathered from 450 total individually collected HIV RNA-negative plasma specimens across eighteen runs with three kit/M1 Lysis Diluent lot combinations, a detection cutoff of 29 copies/mL was defined to deliver specificity with a lower level of the 95% confidence interval of at least 95% (Table 1, Negative cutoff determination). Specificity with the defined detection cutoff was verified as >95% by 144 additional individual HIV-negative plasma specimens across twelve more runs with the remaining three kit/M1 Lysis Diluent lot combinations (Table 1, Negative cutoff verification). The verified detection cutoff was then used in analyzing all other experiments.

**Table 1:** Specificity with individual HIV-negative clinical specimens: determination of 29 copies/mL as the negative cutoff and subsequent verification in the modified assay.

Negative cutoff determination			Negative cutoff verification		
N	N below negative cutoff	Specificity (95% Lower Confidence Limit)	N	N below negative cutoff	Specificity (95% Lower Confidence Limit)
450	436	96.9% (95.2%)	144	140	97.2% (93.8%)

Wells across all thirty runs containing dilution series replicates were used to determine analytical sensitivity, linearity, accuracy, and precision for the modified assay. The assay detection cutoff ascertained from the individual negative specimens was used in determining the assay limit of detection (LoD, 95% detection rate) as 70 copies/mL (95% CI from 64 to 76 copies/mL) by logistic curve fit. Linearity of the modified assay was evaluated by determining the absolute deviation of geometric mean quantitations from the predicted values on the linear regression line for dilution series levels above the LoD. These mean differences for all levels of the dilution series above the LoD were ≤0.07 log_10_ (Table 2, Log Difference). Accuracy of the modified assay was determined from the difference in measured and expected mean quantitations for dilution series levels. These differences were ≤0.06 log_10_ for all levels above the assay LoD with the modified assay (Table 2, Log Recovery). The range of total percent coefficients of variation (%CVs) corresponding to dilution series levels above the LoD in the modified assay were 13.8–31.4% for the modified assay (Table 2). Analytical sensitivity, linearity, accuracy, and precision determined with the modified assay are comparable to the analogous parameters previously reported [2] for the Versant Assay.

**Table 2:** Analytical performance summary for the modified HIV-1 bDNA assay.

Level (copies/mL)	Total N	Positive N	Log Recovery	Log Difference	Total %CV
584926	197	197	0.06	0.06	16.6
58493	197	197	0.01	0.01	20.3
5849	199	199	0.06	0.07	13.8
585	196	196	0.01	0.01	31.3
117	347	345	−0.05	−0.04	30.4
88	347	341	−0.06	−0.05	31.4
58	348	313	−0.04	−0.03	29.7

**Figure 4:**
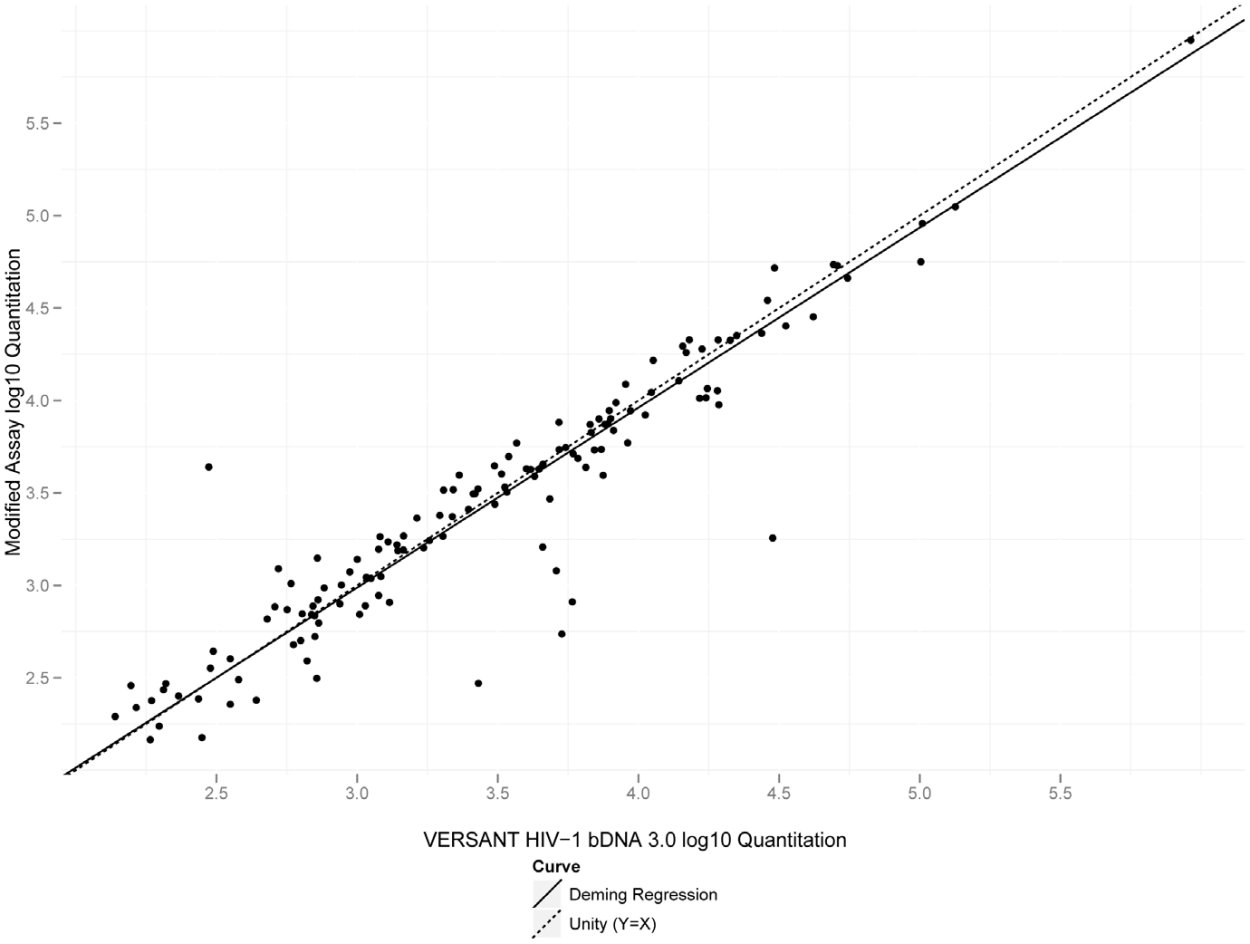
Method comparison of the modified bDNA assay and the VERSANT HIV-1 bDNA 3.0 Assay. Log quantitations of 135 HIV-positive clinical specimens with the modified assay were measured and plotted. The Deming regression line (slope = 0.974 with 95% CI from 0.913 to 1.04) is indicated in the plot.

**Figure 5:**
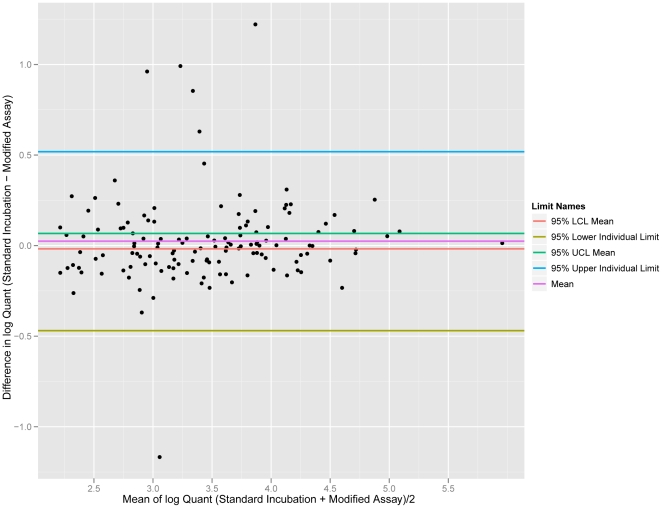
Bland-Altman plot of method comparison data between the modified bDNA assay and the VERSANT HIV-1 bDNA 3.0 Assay. No evidence of proportional bias across the linear range of the assay is apparent (regression slope = −0.026; 95% CI from −0.086 to 0.035).

Quantitative equivalence of the modified assay and the Versant Assay was established with a set of common clinical specimens quantified in both assays (Fig. 4). Results quantifying below the LoD of the modified assay (6 results) were excluded from analysis. Average log difference between the Versant and modified assays was −0.025 (95% CI from −0.067 to 0.018), indicating no statistically significant difference in quantification between the two assays. Deming regression showed linearity for a plot of log quantifications for the Versant Assay against the log quantifications of the modified assay across the range examined (slope = 0.974 with 95% CI from 0.913 to 1.04). A Bland-Altman plot (Fig. 5) demonstrated no evidence of proportional bias across the linear range of the assay (regression slope = −0.026; 95% CI from −0.086 to 0.035).

Genotype equivalence was demonstrated with a high concentration series (∼50,000 copies/mL) and a low concentration series (∼500 copies/mL) of HIV subtype samples. The modified assay and the Versant Assay quantified each sample very similarly (Figs. S1 and S2).

Like the Versant Assay [2], none of the endogenous substances tested for potential interference in the modified assay had a significant effect on assay specificity with HIV-negative samples or quantification of target at approximately 1,000 copies/mL in Seracon II (Table S3).

## Discussion

The modified bDNA assay demonstrated performance characteristics comparable to the more time-consuming Versant HIV-1 RNA 3.0 Assay (bDNA). The dramatically shortened target incubation was enabled by modification of only the Lysis Diluent used in target incubation. The assay retains advantages of bDNA technology, including superior reproducibility, reliability, and insusceptibility to contamination. Throughput with the modified assay is comparable to analogous PCR-based assays used in clinical laboratories. A single Versant 440 Molecular System can run two 96 well plates simultaneously, allowing the modified HIV-1 assay to routinely process 192 tests per day. bDNA assays with a single day turnaround may be ideal for laboratories with especially stringent cost, contamination, or reliability requirements.

Similar shortened target incubation can be implemented in bDNA assays targeting a variety of clinically relevant nucleic acid analytes. The HIV analyte targeted in the viral load assay presented here has a relatively stringent sensitivity requirement. The target incubation time of a bDNA assay can most likely be further shortened when targeting analytes lacking acute sensitivity requirements.

## Materials and Methods

### Assay Kits and Systems

The modified assay used Versant Assay kits, removing the original Lysis Diluent component and replacing it with M1 Lysis Diluent. All other Versant Assay reagents were used unchanged. Assays were performed on the VERSANT® 440 Molecular System with software modified to allow a target incubation period of 2.5 h. Some preliminary experimentation used in developing the M1 Lysis Diluent formulation was performed on the older System 340 bDNA Analyzer. Unmodified kit calibrators and controls from the Versant Assay were used in the modified assay to generate a standard curve and verify plate validity.

### Reagent Modification

In M1 Lysis Diluent, salt and surfactant concentrations were doubled relative to the Lysis Diluent in the Versant Assay. Concentrations of the remaining components of the Lysis Diluent were identical in M1 Lysis Diluent. Additionally, polyvinylsulfonic acid was added to M1 Lysis Diluent (0.3% (w/v)). The target incubation solution volume in the modified assay was reduced to 80 µL from 140 µL per well in the Versant Assay, by adding 60 µL, instead of 120 µL, of “Lysis Working Reagent” made from M1 Lysis Diluent (Table S2).

### Analytical Performance Evaluation

Analytical sensitivity, specificity, linearity, accuracy and precision were evaluated by testing three M1 Lysis Diluent lots made from diverse raw materials in all six permutations available with two assay kit lots. Specificity testing was performed on individual HIV-negative plasma specimens (ProMedDx). A dilution series of non-infectious HIV-1_8E5/LAV_ virus (8E5) [9] in Seracon II (SeraCare) was gravimetrically prepared. The 8E5 stock was previously value assigned from a reference transcript quantified by phosphate analysis [11]. The dilutions were then value assigned in the Versant Assay. This is identical to the dilution series preparation in a published evaluation of the Versant Assay [2], except 8E5 was substituted for β-propiolactone-treated virus. The dilution series was used for all analytical performance testing. Dilution series observations in analytical performance studies with Studentized residuals >4 for relative light unit signal were considered outliers and excluded from analysis (5 of 970 results).

### Method Comparison

In the method comparison experiment, aliquots from 141 HIV-positive clinical specimens (ProMedDx, Bio Collections, or Northwest Biomedical) with viral loads distributed across the assay range were quantified in the Versant Assay and the modified assay. These specimens were collected with written informed consent of the participants and following protocols approved by New England IRB (ProMedDx), Independent Investigational Review Board, Inc. (BioCollections), or Beijing Youan Hospital IRB (Northwest Biomedical). The specimens were purchased delinked and deidentified from the participants by the commercial sources. An unweighted Deming regression was used in the method comparison analysis [12]. No outlier analysis was performed on the method comparison data.

### Genotype Equivalence and Potentially Interfering Endogenous Substances

A panel of HIV RNA-positive samples composed of eight HIV-1 subtypes (SeraCare) was used to examine genotype quantification equivalence. Two levels of each panel member (∼50,000 and ∼500 copies/mL) were prepared by gravimetric dilution in Seracon II. Elevated levels of several endogenous substances, including hemoglobin (200 mg/dL), triglycerides (2000 mg/dL), albumin (6 g/dL), γ-globulins (6 g/dL), and bilirubin (20 mg/dL), were tested for potential interference in the modified assay. These levels are derived from recommended test levels in CLSI document EP7-A2 [13]. The endogenous substance samples were prepared in Seracon II, with 8E5 gravimetrically spiked to create HIV-positive samples.

## Supporting Information

Figure S1 **Eight HIV subtypes at ∼50,000 copies/mL quantified by the modified assay and the VERSANT HIV-1 bDNA 3.0 Assay.** Quantification by the VERSANT HIV-1bDNA 3.0 Assay (32 replicates of each subtype across 4 plates) is very similar to quantification by the modified assay (5 replicates of each subtype on a single plate).
(TIF)

Figure S2 **Eight HIV subtypes at ∼500 copies/mL quantified by the modified assay and the VERSANT HIV-1bDNA 3.0 Assay.** Quantification by the VERSANT HIV-1 bDNA 3.0 Assay (32 replicates of each subtype across 4 plates) is very similar to quantification by the modified assay (5 replicates of each subtype on a single plate).
(TIF)

Table S1 **Analytical performance summary for the unmodified HIV-1 bDNA assay with truncated target incubation.**
(DOC)

Table S2 **HIV Target Incubation: VERSANT versus modified assay volumes.** In the Versant Assay, all reagents (except M1 Lysis Diluent) in Table S3 are supplied in separate vials. The components are combined following the instructions for use provided with the Versant Assay to make a “Lysis Working Reagent”, which is added (120 µL) to each sample well (total incubation volume 140 µL). In the modified assay, M1 Lysis Diluent (45 µL) is substituted for Lysis Diluent and the resulting Lysis Working Reagent is added (60 µL) to each sample well (total incubation volume 80 µL).
(DOC)

Table S3 **Endogenous substances: specificity with HIV-negative samples and quantification with HIV-positive samples in the modified assay.**
(DOC)
