# Supplementary figures and images for: A Sensitive Branched DNA HIV-1 Signal Amplification Viral Load Assay with Single Day Turnaround
Source: PLoS One. 2012 Mar 27;7(3):e33295. doi: 10.1371/journal.pone.0033295 (PMC3314011; doi:10.1371/journal.pone.0033295)

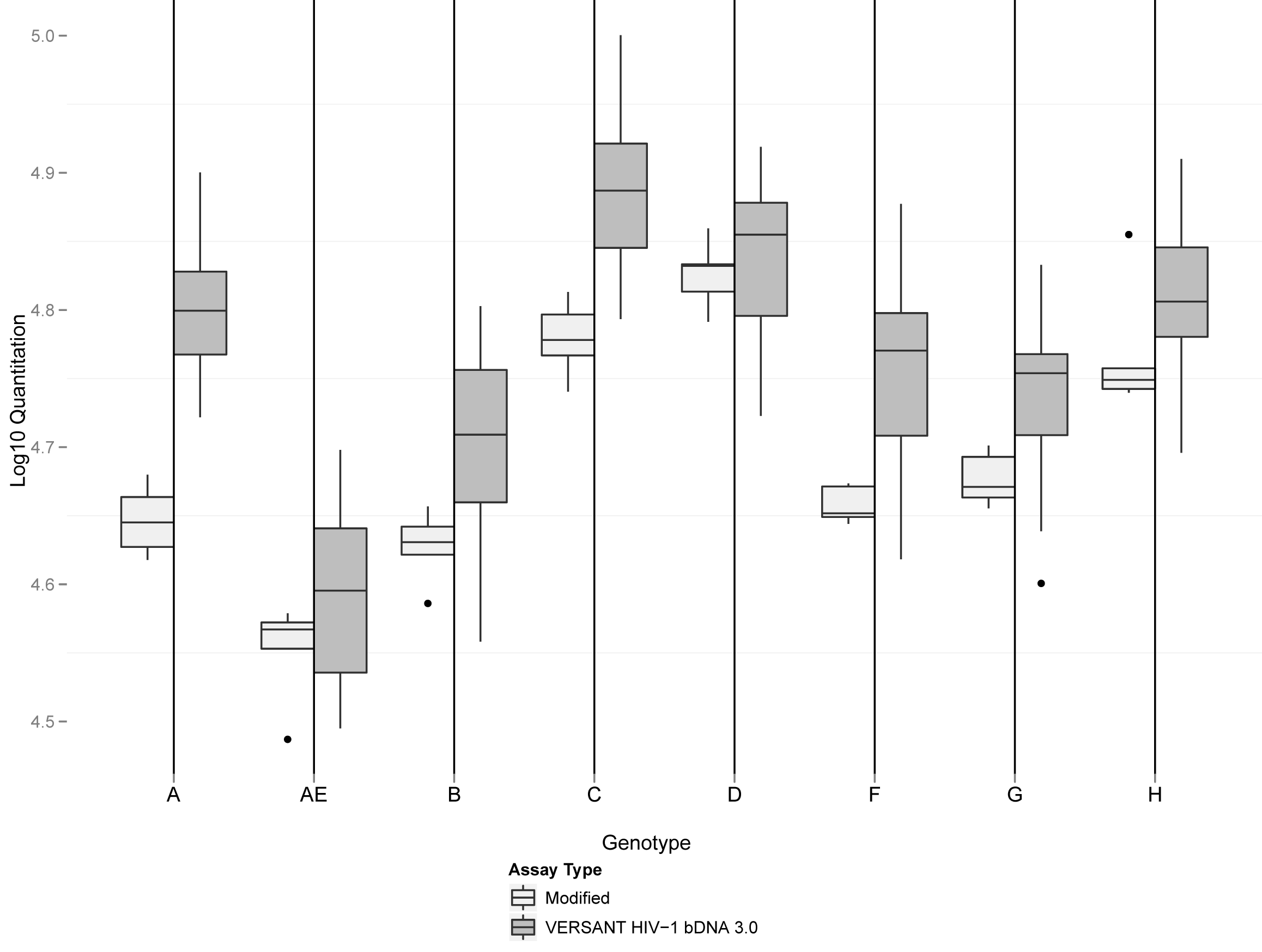

Supplement: Figure S1 — Eight HIV subtypes at ∼50,000 copies/mL quantified by the modified assay and the VERSANT HIV-1 bDNA 3.0 Assay. Quantification by the VERSANT HIV-1bDNA 3.0 Assay (32 replicates of each subtype across 4 plates) is very similar to quantification by the modified assay (5 replicates of each subtype on a single plate). (TIF) [file pone.0033295.s001.tif]

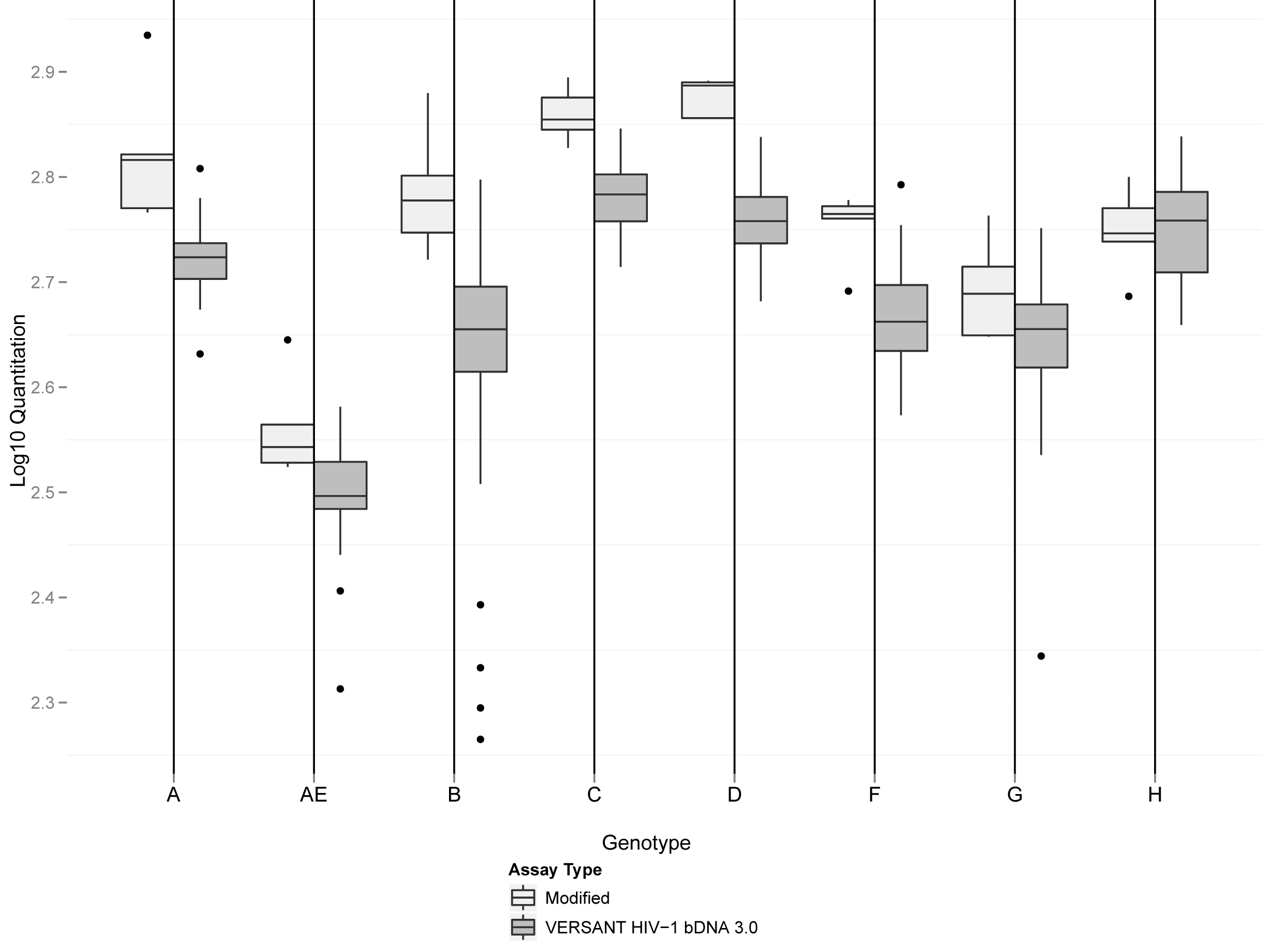

Supplement: Figure S2 — Eight HIV subtypes at ∼500 copies/mL quantified by the modified assay and the VERSANT HIV-1bDNA 3.0 Assay. Quantification by the VERSANT HIV-1 bDNA 3.0 Assay (32 replicates of each subtype across 4 plates) is very similar to quantification by the modified assay (5 replicates of each subtype on a single plate). (TIF) [file pone.0033295.s002.tif]
