# Supplementary material for: A Sensitive Branched DNA HIV-1 Signal Amplification Viral Load Assay with Single Day Turnaround
Source: PLoS One. 2012 Mar 27;7(3):e33295. doi: 10.1371/journal.pone.0033295 (PMC3314011; doi:10.1371/journal.pone.0033295)
Supplement: Table S1 — Analytical performance summary for the unmodified HIV-1 bDNA assay with truncated target incubation. (DOC) [file pone.0033295.s003.doc]

Table S1. Analytical performance summary for the unmodified HIV-1 bDNA assay with truncated target incubation

| **Level (copies/mL)** | **Total N** | **Positive N** | **Log Recovery** | **Log Difference** | **Total %CV** |
| --- | --- | --- | --- | --- | --- |
| 571182 | 72 | 72 | 0.01 | 0.02 | 32.14 |
| 57118 | 72 | 72 | -0.03 | -0.01 | 44.24 |
| 5712 | 72 | 72 | 0.02 | 0.04 | 47.19 |
| 571 | 72 | 72 | -0.11 | -0.09 | 59.10 |
| 114* | 117 | 36 | 0.03 | 0.04 | 33.79 |
| 86* | 117 | 18 | 0.16 | 0.18 | 42.03 |
| 57* | 120 | 1 | 0.26 | 0.28 | 26.56 |

*Below the estimated assay LoD of 149 copies/mL
