# Supplementary material for: A Sensitive Branched DNA HIV-1 Signal Amplification Viral Load Assay with Single Day Turnaround
Source: PLoS One. 2012 Mar 27;7(3):e33295. doi: 10.1371/journal.pone.0033295 (PMC3314011; doi:10.1371/journal.pone.0033295)
Supplement: Table S2 — HIV Target Incubation: VERSANT versus modified assay volumes. In the Versant Assay, all reagents (except M1 Lysis Diluent) in Table S3 are supplied in separate vials. The components are combined following the instructions for use provided with the Versant Assay to make a “Lysis Working Reagent”, which is added (120 µL) to each sample well (total incubation volume 140 µL). In the modified assay, M1 Lysis Diluent (45 µL) is substituted for Lysis Diluent and the resulting Lysis Working Reagent is added (60 µL) to each sample well (total incubation volume 80 µL). (DOC) [file pone.0033295.s004.doc]

Table S2. HIV Target Incubation^1^: VERSANT versus modified assay volumes

|  | **Reagents** | | | | | **Sample** | **Total Incubation** |
| --- | --- | --- | --- | --- | --- | --- | --- |
| **Assay** | **M1 Lysis Diluent^2^** | **Lysis Diluent** | **Lysis Reagent** | **Target Probes^3^** | **Capture Probes^4^** |
| VERSANT | -- | 105 | 13.5 | 0.75 | 0.75 | 20 | 140 |
| Modified | 45 | -- | 13.5 | 0.75 | 0.75 | 20 | 80 |

1. Volumes are μL amounts added to a single well.
2. Concentrations of salt and surfactant were doubled in M1 Lysis Diluent relative to concentrations in the Lysis Diluent.
3. Target Probes initiate construction of an extended signaling structure.
4. Capture Probes immobilize target.

In the Versant Assay, all reagents (except M1 Lysis Diluent) in Table S2 are supplied in separate vials. The components are combined following the instructions for use provided with the Versant Assay to make a “Lysis Working Reagent”, which is added (120 μL) to each sample well (total incubation volume 140 μL). In the modified assay, M1 Lysis Diluent (45 μL) is substituted for Lysis Diluent and the resulting Lysis Working Reagent is added (60 μL) to each sample well (total incubation volume 80 μL).
