# Supplementary material for: A Sensitive Branched DNA HIV-1 Signal Amplification Viral Load Assay with Single Day Turnaround
Source: PLoS One. 2012 Mar 27;7(3):e33295. doi: 10.1371/journal.pone.0033295 (PMC3314011; doi:10.1371/journal.pone.0033295)
Supplement: Table S3 — Endogenous substances: specificity with HIV-negative samples and quantification with HIV-positive samples in the modified assay. (DOC) [file pone.0033295.s005.doc]

Table S3. Endogenous substances: specificity with HIV-negative samples and quantification with HIV-positive samples in the modified assay

|  |  | **Negative Samples** | | **Positive Samples** | | |
| --- | --- | --- | --- | --- | --- | --- |
| **Spiked Substance** | **Concentration** | **N** | **Positive N^1^** | **N** | **Average Log Quantitation**  **(copies/mL)** | **Average Log Quantitation Difference from Control** |
| None | NA | 12 | 0 | 12 | 3.082 | 0 |
| Hemoglobin | 200 mg/dL | 12 | 0 | 12 | 3.052 | -0.0303 |
| Triglycerides | 2000 mg/dL | 12 | 1 | 12 | 3.019 | -0.0629 |
| Albumin | 6 g/dL | 12 | 0 | 12 | 3.092 | 0.0100 |
| γ-Globulins | 6 g/dL | 12 | 0 | 12 | 3.085 | 0.0032 |
| Bilirubin | 20 mg/dL | 12 | 0 | 12 | 3.008 | -0.0736 |

1. One sample was positive, giving an overall specificity of 98.6% for this experiment. This specificity is well within the assay’s acceptable range (>95%). No obvious decrease in specificity in the presence of any of the spiked substances was observed.
